# Small Interfering RNA Effectively Inhibits the Expression of SARS Coronavirus Membrane Gene at Two Novel Targeting Sites

**DOI:** 10.3390/molecules15107197

**Published:** 2010-10-18

**Authors:** Ying Wang, Ying-Li Cao, Fan Yang, Yun Zhang, Shu-Hui Wang, Li Liu

**Affiliations:** Department of Microbiology, Institute of Basic Medical Sciences, Chinese Academy of Medical Sciences & School of Basic Medicine, Peking Union Medical College, Beijing 100005, China; E-Mails: fish_wangying@sina.com (Y.W.).; caoyl2000@sina.com (Y.-L.C.).; yzhang222@sina.com (Y.Z.).; shwangabc@yahoo.com (S.-H.W.)

**Keywords:** siRNA, SARS-CoV, membrane gene, RT-PCR, EGFP

## Abstract

Small interfering RNA (siRNA) is a class of duplex RNA molecules of 21-25 nt nucleotides in length functioning post-transcriptionally to downregulate targeted gene expression. The membrane (M) protein of severe acute respiratory syndrome-associated coronavirus (SARS-CoV) is highly abundant during viral infections and is a critical element for viral assembly. Nucleotide substitution in the viral genome occurs frequently during SARS-CoV infection. In the current study, we analyzed the M gene sequences derived from 15 SARS-CoV isolates and uncovered six nucleotide substitutions among these isolates. Interestingly, these nucleotide substitutions are all located at the 5’ half of the M gene. Based on this information and previous reports, we created two novel siRNAs targeting two unexplored and well conserved regions in the M gene. The effects of these two siRNAs were tested by semi-quantitative RT-PCR and EGFP-M fusion gene expression. The results demonstrated that both siRNAs effectively and specifically blocked the targeted gene expression. Real time quantitative RT-PCR (qRT-PCR) revealed that siRNA targeting the 3’ half of the M gene (si-M2) induced more potent inhibition than that targeting the 5’ half (si-M1). Both si-M1 and si-M2 significantly downregulated M gene mediated upregulation of interferon β expression. Thus, our results indicate that SARS-CoV M gene specific siRNA might function in a sequence-dependent manner.

## 1. Introduction 

Severe acute respiratory syndrome-associated coronavirus (SARS-CoV) which belongs to the *Coronaviridae* family in the *Nidovirales* order, is the largest RNA virus (positive stranded) with a genome size of around 30 kb [1]. Although SARS-CoV is evolutionally distinct from other coronaviruses, the viral genes encoded by all coronaviruses, as well as their gene organization, are all similar. The main gene order from 5’ to 3’ direction is ORF1ab, spike (S), envelope (E), membrane (M), and nucleocapsid (N) proteins [2]. The 5’-most two-thirds of the genome contains two large and overlapped open reading frames, ORF1a and ORF1b (ORF1ab), whose products are two polyproteins that can be further cleaved by virally encoded proteases [1]. ORF1ab encodes several viral regulatory proteins that are essential for viral transcription and replication [1]. SARS-CoV has a lipid bilayer envelope with the multiple insertions of S, M and E proteins [3]. Inside the viral envelope, N proteins are associated with viral genomic RNA to form a helical nucleocapsid structure [3]. In addition, eight group-specific or accessory genes are interspersed between these main structural genes in the 3’-most one-third of the SARS-CoV genome, there are two (3a and 3b) between S and E, five (6, 7a, 7b, 8a, 8b) between M and N and one (9b) in N gene [2,4].

The M protein of SARS-CoV is a triple-spanning transmebrane protein and serves as one of the key elements for viral assembly. The assembled SARS-CoV virions then subsequently bud into the lumen of the endoplasmic reticulum-Golgi intermediary compartment (ERGIC) [5]. The results concerning the minimal requirement for the assembly of viral like particle (VLP) are still controversial. Studies indicated that either M and N or M and E are sufficient for the formation and release of VLPs [6,7]. Differently, Siu *et al*. showed that N and E must co-express with M for the efficient production and release of VLP [8]. In viral envelope, M protein laterally interacts with the other two transmembrane proteins E and S [9]. A 12~24 amino acid element located at the C-terminal domain of M exposes to cytosol and physically interacts with N protein during viral packaging [10,11]. 

M is the most abundant viral protein that may greatly contribute to viral induced pathogenesis by interfering intracellular signaling pathways. For example, SARS M protein inhibits the nuclear factor kappa B (NF-κB) signaling pathway through a direct contact with the upstream kinase IKKβ [12]. SARS-CoV M protein could also inhibit dsRNA-induced interferon β production by interfering with the formation of TRAF3.TANK.TBK1/IKKε complex [13]. The blockade of these signaling pathways may severely impair the host innate and/or adaptive immune responses to SARS-CoV infection. Therefore, inhibition on M gene expression might be a good strategy for anti-SARS drug development. 

Small interfering RNA (siRNA) provides a powerful and specific means to down-regulate targeted gene expression. The M gene specific siRNAs have been screened and identified by a number of groups [14,15,16]. However, the frequent nucleotide substitutions in M gene may potentially release the inhibitory effect induced by these siRNAs. In the current study, we compared and analyzed the M gene sequences derived from 15 different SARS-CoV isolates. Two novel siRNAs targeting at the conserved and unexplored regions in M gene were tested for their inhibition on M gene expression. The results of current study may provide valuable information for the design of more effective siRNA against the M gene of SARS-CoV. 

## 2. Results and Discussion

### 2.1. The selection of the targeted sites for M gene specific siRNAs

**Figure 1:**
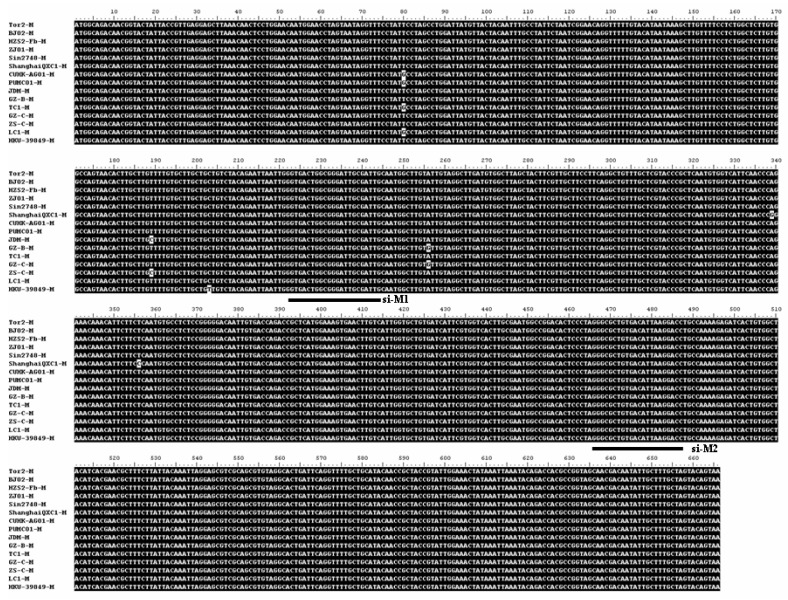
Sequence alignment of the M genes of different SARS-CoV isolates. Two novel siRNAs (si-M1 and si-M2) targeting at 221~242nt and 466~486nt, respectively, are underlined.

SARS-CoV is a positive stranded RNA virus with higher mutation rates in the viral genome [17]. A better siRNA should be designed to target the conserved region of the targeted gene. Therefore, we compared the M genes derived from 15 isolates of SARS-CoV. These isolates were Tor2 (AY274119), BJ02 (AY278487), HZS2-FB (AY394987), ZJ01 (AY297028), Sin2748 (AY283797), ShanghaiQXC1 (AY463059), CUHK-AG01 (AY345986), PUMC01 (AY350750), JDM (AY394988), GZ-B (AY394978), TC1 (AY338174), GZ-C (AY394979), ZS-C (AY95003), LC1 (AY394998.1) and HKU-39849 (AY278491). Sequence alignment revealed that there were six nucleotide substitutions in the M genes among these isolates (Figure 1). Interestingly, all six nucleotide substitutions (nt80, 189, 203, 256, 339 and 356) are located within the 5’ half (first 360nt) of M gene (total 666nt), indicating that higher mutations are associated with the 5’ portion of the M gene. 

Previously, a number of M gene siRNAs based on random selection have been reported [14,16,18]. Considering the nucleotide substitutions in M gene as well as the previous reports, we chose two unexplored regions that were well conserved in the M genes among the 15 isolates of SARS-CoV. One targeting site was at +221~+242nt and the other at +466~+486nt relative to the 5’ ATG initiation codon (Figure 1). 

### 2.2. Cloning and expression analysis of M gene

**Figure 2:**
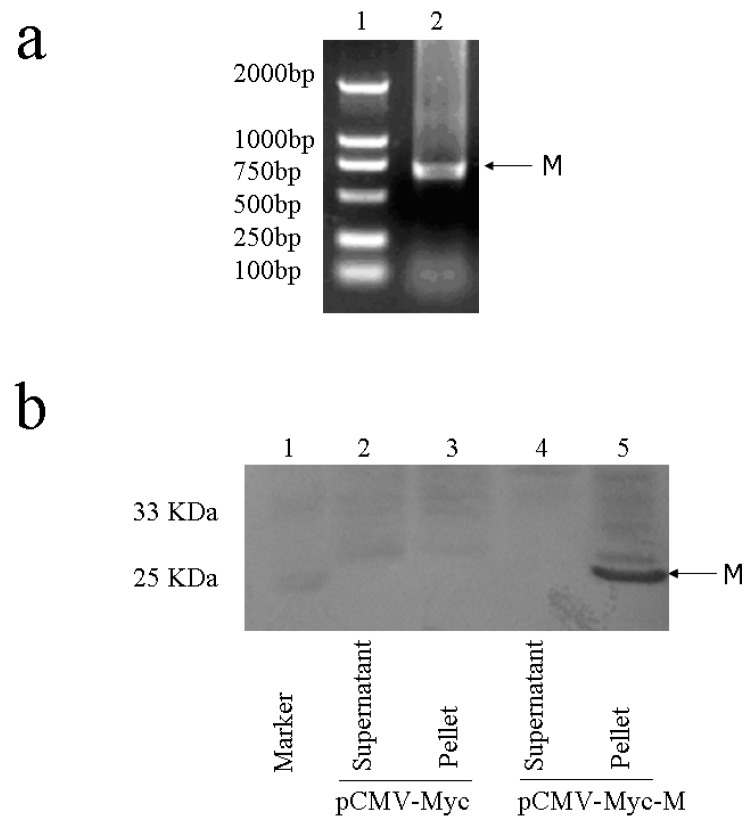
Cloning and expression analysis of SARS-CoV M gene. (a) M gene was cloned by RT-PCR. (b) Western blot analysis on M gene expression in HEK293T cells. HEK293T cells were transfected with either pCMV-Myc or pCMV-Myc-M. After 48 h, the transfected cells were lysed and subjected to brief centrifugation to separate supernatant from cell pellet. Equal amount of cell supernatant and pellet were resolved onto 12% SDS-PAGE. The reaction products were probed with anti-Myc antibody.

The full length of SARS-CoV M gene was amplified from Vero E6 cells infected with the viral strain HKU-39849 (Figure 2a). The complete coding sequence of M gene was then subcloned into eukaryotic expression vector pCMV-Myc to generate plasmid pCMV-Myc-M. Western blot analysis indicated that SARS-CoV M protein was insoluble. The result showed that no significant amount of M proteins was presented in the supernatant fraction, while a large amount of M protein could be readily detected in the cell pellets (Figure 2b). The result is in agreement with a previous report that SARS-CoV M proteins can be thermally aggregated upon heat treatment [19]. 

### 2.3. Two novel siRNAs effectively inhibits M gene expression

**Figure 3:**
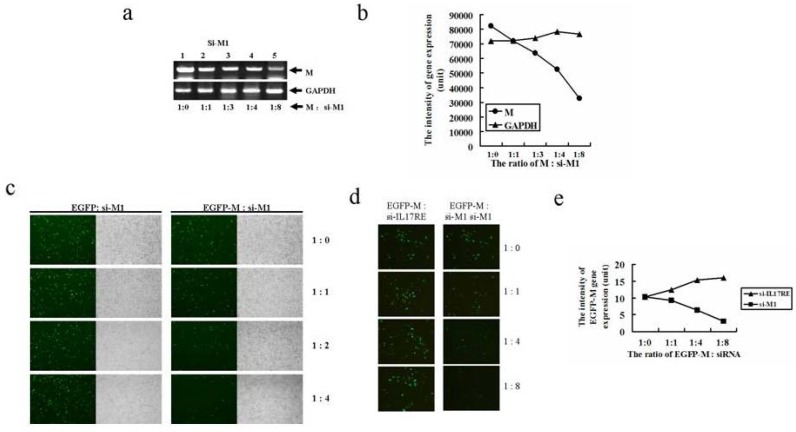
The inhibitory effect of siRNA1 on SARS-CoV M gene expression. (a)RT-PCR analysis on si-M1 mediated M gene repression. About 1μg of pCMV-Myc-M was co-transfected with increased doses of si-M1. The reaction products were subjected to RT-PCR analysis using SARS-M and GAPDH primers. (b) Quantitation of the RT-PCR results in (a). (c) si-M1 specifically inhibited pEGFP-M fusion gene but not pEGFP gene expression. About 1μg of either pEGFP-M or pEGFP was co-transfected with increased doses of si-M1. (d) si-M1 but not si-IL-17RE effectively inhibited EGFP-M gene expression. About 1μg of pEGFP-M plasmid was co-transfected with increased doses of either si-M1 or si-IL17RE. (e) Flow cytometric analysis on the intensity of pEGFP-M gene expression in (d).

We designed two siRNAs specifically targeting these two regions as shown in Figure 1, and named them si-M1 and si-M2, respectively. M gene was also fused with the EGFP gene to make the pEGFP-M construct. The effect of siRNA on M gene expression was monitored by both RT-PCR and EGFP expression. Figure 3a demonstrated a significant reduction in M gene expression as the ratio of M to si-M1 reaching 1:8. Quantitation of the band intensity revealed about a 2~3 fold reduction in M mRNAs as co-transfecting with higher doses of si-M1 (Figure 3b). Moreover, a marked inhibition on EGFP-M, but not EGFP expression was observed when administrated with higher doses of si-M1 (Figure 3c). The specificity of si-M1 on M gene expression was further confirmed by using a non-specific siRNA, si-IL17RE, as a negative control. Higher doses of si-M1 but not si-IL-17RE effectively inhibited EGFP-M gene expression (Figure 3d). Flow cytometric analysis indicates that the intensity of EGFP-M gene expression was significantly inhibited by si-M1 but not si-IL17RE as the delivered dose increased (Figure 3e). 

**Figure 4:**
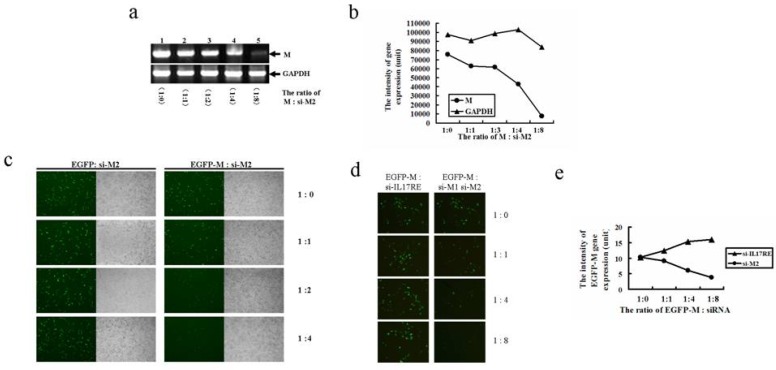
The inhibitory effect of siRNA2 on SARS-CoV M gene expression. (a) RT-PCR analysis on the dose effect of si-M2 on M mRNA expression. About 1 μg of pCMV-Myc-M was co-transfected with increased doses of si-M2. The reaction products were subjected to RT-PCR analysis using SARS-M and GAPDH primers. (b) Quantitation of the RT-PCR results in (a). (c) si-M2 specifically inhibited pEGFP-M fusion gene but not pEGFP gene expression. About 1 μg of either pEGFP-M or pEGFP was co-transfected with increased doses of si-M2. (d) si-M2 but not si-IL-17RE effectively inhibited pEGFP-M gene expression. About 1 μg of pEGFP-M plasmid was co-transfected with increased doses of either si-M2 or si-IL17RE. (e) Flow cytometric analysis on the intensity of pEGFP-M gene expression in (d).

Similarly, si-M2 which targeted at the 3’ half of M gene also dramatically inhibited M mRNAs by about 7~8 fold when the molar ratio of si-M2:M was increased to 8:1 (Figure 4a and Figure 4b). si-M2 mediated EGFP gene inhibition was also markedly induced when a higher dose of si-M2 was co-transfected with EGFP-M fusion gene but not with EGFP gene (Figure 4c).

Higher doses of si-M2 but not si-IL-17RE effectively inhibited EGFP-M gene expression (Figure 4d). Flow cytometric analysis indicates that the intensity of EGFP-M gene expression was significantly inhibited by si-M2 but not si-IL17RE as the delivered dose increased (Figure 4e). Overall, the above results clearly demonstrated that both si-M1 and si-M2 are effective inhibitors to block SARS-CoV M gene expression. 

### 2.4. siRNA targeting the 3′ portion of M gene is a more potent inhibitor

**Figure 5:**
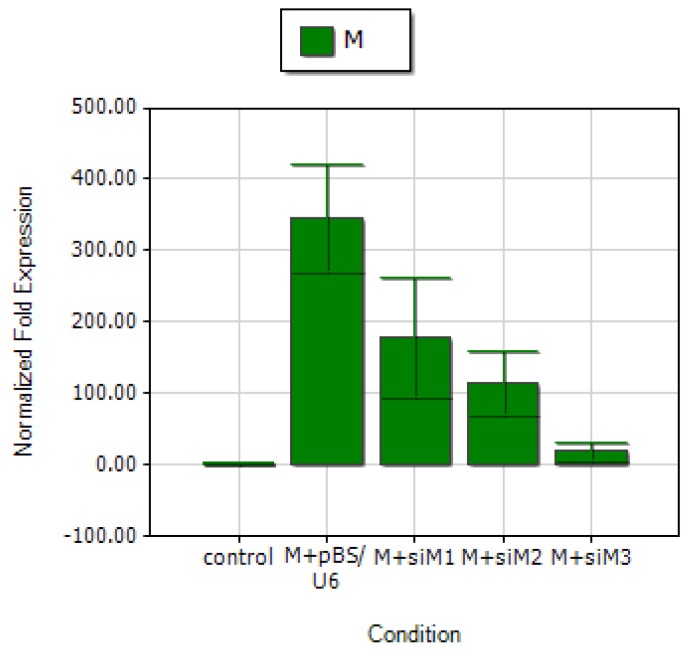
Comparison of the inhibitory effect mediated by si-M1, si-M2 and si-M3 on M gene expression by qRT-PCR analysis. About 2 μg of M gene was co-transfected with 4 μg of each plasmid pBS/U6, si-M1, si-M2 and si-M3, while co-transfection of 2 μg of pCMV-Myc plus 4 μg of pBS/U6 was served as negative control. Both β-actin-R and SARS-M-R primers were used in this analysis.

Previous studies showed that specific siRNAs targeting at the terminal sequences of M coding region are more potent gene silencers [15,16]. To assess the strength of gene inhibition induced by si-M1 and si-M2, as well as those known siRNAs, real time qRT-PCR was employed to directly measure siRNA-mediated M gene repression that included the siRNA targeting the 3’ terminus (637-657nt) of M gene (named as si-M3 in this study) described by Qin *et al*. [15] as a positive control. Both si-M1 and si-M2 were effective to down-regulate M gene expression but with a less effect than that of si-M3 (Figure 5). Targeting at the 3’ portion of M gene by si-M2 and si-M3 generated more potent inhibition on M mRNA expression than that of si-M1 (Figure 5). Our study indicates that siRNA duplex mediated gene silencing in SARS M gene expression might work in a sequence-dependent manner. 

### 2.5. M gene specific siRNAs effectively inhibited M gene mediated upregulation of interferon β gene expression

**Figure 6:**
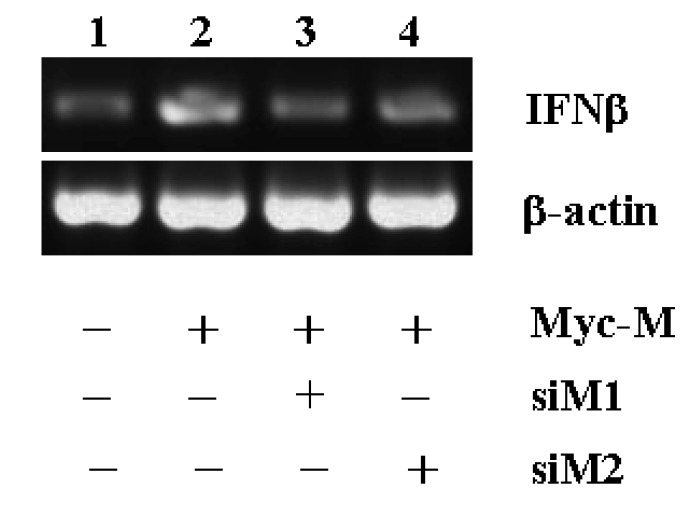
The counteractive effect of si-M1 and siM2 on M gene mediated interferon β production. HEK293 cells were co-transfected with 2 μg plasmid pCMV-Myc-M plus 4 μg of each pBS/U6 (lane 2), pBS/U6-siM1 (lane 3) or pBS/U6-siM2 (lane 4). Co-transfection of 2 μg pCMV-Myc plus 4 μg of pBS/U6 was served as negative control (lane 1). About 1 μg of total RNA isolated from each reaction was subjected to RT-PCR analysis using either IFNβ or β-actin primers.

The interferon β production is usually induced by dsRNA [20]. However, we found that overexpression of M gene alone in HEK293 cells was able to induce IFNβ production (Figure 6, lane 1 and 2). The IFNβ induction by M gene could be reversed by M gene specific siRNAs. Either si-M1 or si-M2 could effectively downregulate M gene mediated IFNβ production (Figure 6), indicating that both si-M1 and si-M2 were functionally effective to repress SARS-CoV M gene expression. 

## 3. Experimental 

### 3.1. Cell culture and antibodies

Human embryonic kidney cell line 293 (HEK293) and SV40 T antigen transformed HEK293 (HEK293T) were derived from the Cell Culture Center of Institute of Basic Medical Sciences, Chinese Academy of Medical Sciences. Cells were cultured in Dulbecco’s Modified Eagle Medium (HyClone, South Logan, UT) supplemented with 10% fetal calf serum and incubated in a 37 °C incubator containing 5% CO_2_. Anti-Myc antibody was purchased from Santa Cruz Biotechnology (Santa Cruz, CA, USA).

### 3.2. Plasmid construction

The SARS-CoV M gene was amplified from viral strain HKU-39849 (provided by Dr. KY Yuen, The University of Hong Kong) with a pair of primers 5′-tatagaattctggcagacaacggtactatt-3′ and 5′-tataggtaccgtcacttactgtactagcaaagc-3′. After gel purification and restriction endonuclease digestion, the reaction products were subcloned into the EcoRI/KpnI sites of pCMV-Myc to generate pCMV-Myc-M. For construction of M gene specific siRNAs, two novel targeted sites (221nt-242nt and 466nt-486nt) in M gene coding sequence were selected. Four oligos for each targeted site were synthesized as 1a, 5’-ggtgactggcgggattgcgata-3’; 1b, 5’-agcttatcgcaatcccgccagtcacc-3’; 2a, 5′-agcttat cgcaatcccgccagtcaccctttttg-3′; 2b, 5′-aattcaaaaagggtgactggcgggattgcgata-3′ and 1a′,5′-ggcgcgtgacatta aggaca-3’; 1b′, 5′-agcttgtccttaatgtcacagcgcc-3′; 2a′,5′-agcttgtccttaatg tcacagcgccctttttg-3′; 2b′, 5′-aattca aaaagggcgctgtg acattaaggaca-3′. The oligoes 1a and 1b, 2a and 2b, 1a′ and 1b′, 2a′ and 2b′ were annealed pair-wisely to form duplexes. To construct the siRNA targeting to the 3′ terminus of M gene (named as si-M3 that targets to 637-657nt) as described by Qin *et al*. [15], two synthesized oligoes 5′-aacgacaatattgctttgctaa agctttagcaaagcaatattgtcgtttttttg-3′ and 5′-aattcaaaaaaacgacaatattgctttgctaaagc tttagcaaagcaatattgtcgtt-3′ were also annealed. The duplex products were then subcloned into pBS/U6 [21] (kindly provided by Dr. Yang Shi, Harvard Medical School) to form pBS/U6-siM1, pBS/U6-siM2 and pBS/U6-siM3, respectively.

### 3.3. Reverse transcription-polymerase chain reaction (RT-PCR) and real time quantitative RT-PCR (qRT-PCR)

Total RNAs were extracted from the cultured cells with TRIzol (Invitrogen, Carlsbad, CA, USA). All primers used in the RT-PCR reactions were listed in Table 1. One μg of total RNAs was first reverse transcribed using AMV reverse transcriptase (Promega, Madison, WI, USA). About 2 μL of the transcribed cDNAs was subjected to standard PCR reaction using M gene specific primers. One-step real-time quantitative RT-PCR (qRT-PCR) (Takara Biotechnology, Dalian, China) was also performed to monitor the targeted gene expression. Real time qRT-PCR was carried out with iQ5 real-time PCR detection system (Bio-Rad Laboratories, Hercules, CA, USA ) at the following conditions: 42 °C for 5 min and 95 °C for 10 sec; 95 °C for 5 seconds and 60 °C for 10 seconds and repeated for 40 cycles. The dissociation of the reaction products was conducted from 55 °C to 95 °C as the temperature rose at 0.2 °C per ten seconds. 

### 3.4. Transient transfection

Cell cultured in 35-mm dishes were transiently transfected with the indicated plasmid DNAs using ProFection^®^ Mammalian Transfection Systems (Promega) according to the supplier’s instructions. Briefly, transfected DNAs were first mixed with 2M CaCl_2_ (37 μL) and brought to a total volume of 300 μL with sterile and deionized water. Then the DNA-CaCl_2_ mixture was added into equal volume of 2×HBS drop by drop accompanying with gentle vortexing. After 15 minutes incubation, the reaction mixture was evenly distributed into the cell culture medium and incubated for 48 hours before harvesting.

### 3.5. Western blot analysis

The transfected cells were lysed with a lysis buffer containing 1% NP-40, 50 mM Tris-HCl (pH 7.5), 120 mM NaCl, 200 μM NaVO_4_, 1 μg/mL leupeptin, 1 μg/mL aprotinin, and 1 μM PMSF. About 15 μg of cell lysate for each sample was resolved onto 12% SDS-PAGE. After separation, the separated proteins were transferred onto Hybond nitrocellular membrane (Pharmacia, St. Louis, MO, USA). The transferred membrane was first probed with a primary antibody. Then, a secondary antibody labeled with horseradish peroxidase was added to the reaction and finally visualized with an ECL kit (Santa Cruz Biotechnology, Santa Cruz, CA, USA).

### 3.6. Flow cytometric analysis

HEK293 cells were transiently co-transfected with EGFP-M plus either si-M1 or si-M2, while the contransfection of EGFP-M plus si-IL-17RE#1 [22] was served as a negative control. After 48-h incubation, the transfected cells were released from the culture plates and resuspended into 1×PBS. The intensity of EGFP gene expression was measured using the FACSARIA flow cytometer (Becton Dickenson, San Jose, CA, USA). 

## 4. Conclusions

The M protein of SARS-CoV is a key element required for viral assembly. Our data shows that the M protein was not present in the soluble fraction, but can be readily detected in cell pellets, indicating that M protein has the propensity to self-aggregate. 

SARS-CoV M genes were derived from 15 different viral isolates and it was found that nucleotide substitutions were exclusively located at the 5’ half of the M gene (the first 360 nt). Two novel and conserved siRNA target sites were identified and tested. Both siRNAs (si-M1 and si-M2) could effectively inhibit M gene expression post-transcriptionally and functioned in a dose-dependent manner. Moreover, si-M2 targeting the 3’ half of the M gene produced more potent inhibition than that of si-M1 that recognized a conserved sequence at the 5’ half of the M gene. This observation is consistent with a previous report described by Qin *et al.* [15]. The less inhibitory effect induced by si-M1 was correlated with a higher chance of mutation in the targeted region nearby (such as nt203 and nt256). Moreover, our study indicates that both si-M1 and si-M2 were able to functionally counteract the M gene-mediated INFβ production. This study indicates that siRNA mediated M gene inhibition might be determined by the sequence content presented at or around the targeted site. 

## Figures and Tables

**Table 1:** Primers used in RT-PCR analysis.

Gene name	GenBank ID	Forward primer	Reverse primer	Size of product (bp)
*β-actin*^†^ *GAPDH* *INFβ* *SARS-M* ^†^ *β-actin-R*^††^ *SARS-M-R*^††^	BC009275 NM_002046 NM_002176 AY278491 BC009275 AY278491	5′-cacactgtgcccatctacga-3′ 5’-tcttcaccaccatggagaag-3’ 5′-atgaccaacaagtgtctcct-3′ 5′-tatagaattctggcagacaacggtactatt-3′ 5′-tccatcatgaagtgtgacgt-3′ 5′-tgctgtgatcattcgtggtc-3′	5′-ctgcttgctgatccacatct-3′ 5’-ctgcttcaccaccttcttga-3’ 5′-ttcagtttcggaggtaacct-3′ 5′-tataggtaccgtcacttactgtactagcaaagc-3′ 5′-ctcaggaggagcaatgatct-3′ 5′-tacggtagcggttgtatgca-3′	600 489 564 686 161 178

^†^ primers for standard RT-PCR; ^††^ primers for quantitative RT-PCR.
